# Gen’l Tom Thumb

**Published:** 1848-04

**Authors:** 


### GEN'L TOM THUMB.

The levees of this distinguished personage, have, during the last week, been crowded by the good folks of our City. The first few days, the Masonic Hall was a perfect jam, and as character after character was personated by the Lilliputian General, admiration was more and more increased, until, in his splendid Highland costume, he caps the climax of his performance, and wins universal applause. But we are not journalists whose duty it is to present to our readers general intelligence, irrespective of the important interests of our profession—the natural inquiry to the mind of the Dentist is, How does his teeth compare with the balance of the system, symmetrical and finely proportioned as it is? Have these important organs been also arrested in their growth, and present themselves in a corresponding size to the general organization?

Having been called upon professionally to attend to the General's teeth, while in this city, we are prepared to answer these questions somewhat satisfactorily.

In the first place, the sudden check given to the full development of the general organization, has not, in this case at least, put a stop to the development of that set of organs so essential to the preparation of food for the balance of the system. We stop not to admire the wisdom displayed by Providence in even this, for had these organs been arrested in their growth, no adequate masticating apparatus would have been furnished for the comminution of food, which is required for the maintenance of this perfect little gentleman.

The deciduous teeth therefore matured, (we know not if as early as usual,) of the usual size, form and physiological appearance. But nature stopped not even here—although delayed in her operation, yet she is still at work, and that which is destined only for the child in others, also slowly yields to the manhood of the General, hence we find the four upper incisors of the deciduous sett gone, and in their place, as yet, but the two central—these however well developed, of full usual size, and good healthy yellowish color. Below we find the four incisors gone, and their place supplied also by the two central, one of which is at least one half broader than usual, and somewhat resembles a central upper incisor—these two below so fully supplied the place of the four, that to prevent irregularity we had to remove one of the canine, the root of which was but little absorbed, and the extraction of which the General bore with commendable fortitude, showing an amount of resolution, more in proportion to his age than size.

One of the bicuspides below, has been shed, and a permanent one fills its place. The other deciduous teeth still remain, but show evident signs that nature's process is going on for their displacement.

The four anterior permanent molars have made their appearance of full size. These teeth, however, particularly the two in the upper jaw, have been, and are yet, so much embedded in the soft parts as to cause an early decay. This, for want of time, could only receive partial attention. They appear to be much in the same situation as we often find the dens sapientiæ

in the mouth of those grown, and who have scarce room for their admission. Should the other molars and dens sapientiæ, make in time their appearance, we can scarce conceive where they will find room.

In the case before us, we find that nature has pursued her regular course thus far, except in that she moves more slowly—that she will ever complete in full the process of dentition, we can scarce believe. The permanent teeth thus far, are full as large as if designed for a man six feet high instead of twenty-eight inches, and two hundred pounds instead of fifteen.

To the dentist, the history of this case affords food for much contemplation. Why should these organs go on to a full development, and the balance (except indeed the brain) remain as when at seven months old?—CIN. ED.

